# Rupture of the Umbilical Cord, and of the Vagina

**Published:** 1847-06

**Authors:** Wm. Workman


*Rupture of the Umbilical Cord.—Rupture of the Vagina.*—By Wm. Workman, M.D.—On the 20th of October, 1838, I was called to visit Mrs. R., aged about 38, an Irish woman, naturally of robust constitution, but with health somewhat impaired, then in labor with her fifth child. First saw her at 7, P. M. According to her account, she had been in labor about thirty hours; the first part of the time the pains were moderate, but for the last seven or eight hours they had been very urgent and almost incessant. On examination, I found the os uteri well dilated, the membranes protruding into the pelvic cavity and very tense; but the presenting part of the fœtus was high up, yet above the brim of the pelvis. After some fifteen minutes' delay, I ruptured the membranes; and such was the expulsive force of the uterus that it threw a jet of the liquor amnii more than three feet from the bedside. After a few minutes respite the uterine action was renewed as vigorously as before, and the head very soon descended partly into the pelvis; the position being the first oblique, or left acetabular; and I was for a time encouraged by the hope that the labor would be speedily finished by the natural efforts, notwithstanding the obstacles to be overcome.

The opposing difficulties were: 1st, a contraction of the brim of the pelvis, latterally, rendering its transverse and oblique diameters, as I judged, considerably below the medium size; perhaps a rare conformation. The lower strait of the pelvis was of ample dimensions. 2d. A more serious obstruction was occasioned by a globular tumor of from one and a half to two inches in diameter, situated upon the sacrum, just below the promontory. It was firmly attached upon a broad base, immovable, but quite elastic, being a little to the right of the centre, and to the touch communicating the idea of a fibro-cartilaginous structure. At this stage of the case the coronal region was pressing firmly on the tumor, the vertex had descended partly behind the symphsis, and the occiput rested on the left ramus of the pubes. Here it remained two hours, without making the least progress. The elasticity of the tumor would allow the head to advance a little during the height of the pains, but as soon as the action of the uterus slacked, it would recede to the same point again. Meanwhile the patient was vociferous, extremely restless, and most earnestly entreated that I would "use the instruments and relieve her from her torments."

I had delivered her with the forceps in two preceding labors under similar circumstances. with no untoward occurrence, except incontinence of urine for three or four days after the first accouchement. She had borne two children in Boston previously; the first, according to her account, was born by the natural efforts after a somewhat tedious and protracted labor. Of the second she was delivered with the forceps by the late Dr. Doane, of that city, after three days intense suffering, having been reduced to a state of great exhaustion.

Considering all the circumstances of the case, therefore, I was satisfied that longer delay would be useless. The patient lay upon the left side upon a straw bed on the floor — a position not the most convenient for the operator, but as good as circumstances would permit. The left ear could only be reached with much difficulty during the slight remission of the pains, but the forceps were carefully introduced, and in a few minutes so adjusted as to lock readily, though the head was so high up as to carry the junction of the blades within the vulva. As soon as the extraction by the forceps was commenced, the patient exclaimed that her pain was very much relieved, that "she didn't suffer half as much as she did before." It required all the force I could possibly exert during a number of pains, together with the organic and voluntary efforts of the patient, which were neither weak nor slow, before the head could be advanced a single inch; but as soon as it had passed the superior strait, the labor was finished with great facility. While, however, the head was passing the vulva, the perineum still covering the face, I discovered the end of the ruptured umbilical cord hanging loose on the left side in front of the ear, then prolapsed about an inch and a half. I immediately brought out the head, urged the patient to make what voluntary effort she could, introduced my finger under the axilla, and speedily brought out the body of the child; but it was dead—perfectly blanched—exhibiting every appearance of death by hemorrhage shortly before birth.—(The child was a female, of full size ; weight, by estimation, about nine pounds.) The head was scarcely marked by the forceps, which were very accurately applied, the blades passing from the vertex over each ear, one just reaching, and the other falling a few lines short of, the angle of the mouth. The skin was unbroken ; the only injury was a slight cut at the right angle of the mouth on the inside of the upper lip, where it was passed by the point of the blade of the forceps. The cord was ruptured transversely (two thirds its diameter), an inch and a half from the umbilicus; in the other third it ran off obliquely, three fourths of an inch further.

I discovered no prolapsus of the cord until the ruptured extremity appeared by the side of the head, as it was emerging from the external organs. There were no *folds* of it prolapsed, and none about the neck of the fœtus, and, on careful examination, not the least mark of injury or violence of any kind could be detected on any part, except the ruptured extremity ; and this must have been out of the reach of either forceps or fingers when the accident occurred.

I have been puzzled to account for this rupture. In domestic animals, where the cord is forcibly ruptured, it generally takes place some distance from the umbilicus; in the larger animals, from eight to twelve inches or more. If in this case it was occasioned by violence of any kind, why should it not have taken place at the point where that violence was applied? If it had been entangled under the forceps in any way, surely they must have left marks sufficient to show clearly where the force was applied.

I have described this case thus circumstantially and minutely, because it presents two points of considerable interest. And, in order that the result may be duly appreciated, it seemed necessary that the history and treatment should be accurately and fully detailed. Rupture of the umbilical cord is an accident of somewhat rare occurrence ; it may happen without any obvious cause; it may be the result of improper or unnecessary officious interference of the accoucheur.—Judge ye. The pelvic tumor was one of extraordinary character. It was not a bony projection at the top of the sacrum, such as is often found in distortions of the pelvis ; it was less solid than bone, and was of a character essentially different from the recto-vaginal tumors described by authors. This was the main cause of difficulty in the labor at this time, and on the two preceding occasions when I attended her, and probably was the cause of the difficulty in her second accouchement in Boston.

I was called upon to visit this woman again in labor, January 13, 1840, in consultation with three medical gentlemen then in attendance. Meanwhile she had removed to a place some eleven or twelve miles distant from this town. I arrived at her residence at 6 o'clock, A. M., and found the attending physician present; the others had retired. He informed me that she had been in labor about three days, that there was nothing peculiar the first twenty-four hours, the pains were neither severe nor very frequent, and little or no progress was made. During the second day the pains increased, the head of the fœtus presented, advanced into the superior strait, was then arrested, and remained stationary for more than thirty hours. He had noticed the pelvic tumor, which he supposed caused the delay. During all this period the action of the uterus was continued with great force, and with scarcely any remission. About 4 o'clock, P. M., of the day before, while suffering a tremendous pain, she felt something give way in the region of the uterus; the pain immediately ceased, and was only slightly renewed two or three times afterwards. She complained of great prostration, of sinking and faintness at the epigastrium, and of distress diffused over the bowels. There was at this time extreme frequency of pulse, tenderness of the whole abdomen, the countenance had an anxious expression, the lips were swollen and somewhat livid. She begged most earnestly to be delivered without delay.

The attending physician, as he informed me, had suspected a rupture of the uterus, yet he was not fully satisfied of the correctness of that opinion, his colleagues of yesterday not concurring in it. I immediately sat down to the patient, and on examination readily discovered the os uteri, contracted to its usual size after the completion of labor. The vagina was extensively lacerated at its junction with the cervix uteri, and entirely detached from it on the left side and in front.—The fœtus and placenta were in the peritoneal cavity among the intestines. I passed my hand up very carefully along the head, body and extremities of the fœtus, till I reached and felt very distinctly the anterior edge of the liver, in contact with which I found the feet, seized them and brought them down, and at length succeeded in accomplishing the full delivery of the patient, occupying about half an hour in the operation. The child was a male, and, like the previous children, of rather large size. The hemorrhage had been slight, a few small coagula only were found and removed. The patient bore the operation well, and expressed much gratification when it was finished.

The attending physician afterwards informed me that she lived till 4 o'clock, P. M., of same day, twenty-four hours from the time of the rupture. No *post-mortem* examination was made.

*Postscript.*—"There are two causes," says Dr. Blundell, "to which laceration of the uterus may be ascribed; the one is continued resistance to the passing of the fœtus, and the other is obstetric violence, whether of instruments or the hand." The former was obviously the cause in this case.—Whether the life of the mother or child in this case might have been saved by delivery immediately after the occurrence of the rupture, is a question not to be asked. That she might have been safely delivered with the aid of the forceps before this occurrence, is very probable.

Rupture of the uterus or vagina is an obstetrical calamity, of all others, most to be deplored. It generally proves fatal to both mother and child. Yet many instances are recorded by obstetrical writers, of the preservation of one or both.—To this end artificial delivery should be promptly effected. "The practice is, at present," says Dr. Collins, of Dublin, "invariably to effect the delivery as soon as possible after the laceration has taken place." He reports three cases of recovery of the mother, and two children saved. Burns, Dewees, Blundell and Moreau, all decidedly recommend this practice. When the laceration is not extensive, and the fœtus is still retained in utero, the delivery may be effected, by skilful management, either with the forceps or by the perforator and crotchet. In extensive lacerations of the uterus and vagina, or of the vagina alone, the fœtus will generally escape into the peritoneal cavity, and the delivery may generally be accomplished by turning. Dr. Blundell charges his pupils thus, "in these dreadful emergencies." "If you are unequal to the duty, give up the management of the case altogether, and send for further assistance." If the circumstances are such that delivery, *per vias naturales*, is impracticable, an immediate resort to gastrotomy is very decidedly urged by Dewees and Moreau, rather than abandon a patient to her fate, undelivered. "Some few examples attest the success of this method," says Moreau. "It has saved some women; even some children have, through its agency, been born alive."

*Boston Med. and Surg. Journal.*

